# Abrupt termination of the 2019/20 influenza season following preventive measures against COVID-19 in Denmark, Norway and Sweden

**DOI:** 10.2807/1560-7917.ES.2021.26.22.2001160

**Published:** 2021-06-03

**Authors:** Hanne-Dorthe Emborg, AnnaSara Carnahan, Karoline Bragstad, Ramona Trebbien, Mia Brytting, Olav Hungnes, Emma Byström, Lasse S Vestergaard

**Affiliations:** 1 Statens Serum Institut, Copenhagen, Denmark; 2 The Public Health Agency of Sweden, Stockholm, Sweden; 3 Norwegian Institute of Public Health, Oslo, Norway

**Keywords:** influenza, COVID-19, Scandinavian countries, impact preventive measures

## Abstract

**Background:**

In mid-March 2020, a range of public health and social measures (PHSM) against the then new coronavirus disease (COVID-19) were implemented in Denmark, Norway and Sweden.

**Aim:**

We analysed the development of influenza cases during the implementation of PHSM against SARS-CoV-2 in the Scandinavian countries.

**Method:**

Based on the established national laboratory surveillance of influenza, we compared the number of human influenza cases in the weeks immediately before and after the implementation of SARS-CoV-2 PHSM by country. The 2019/20 influenza season was compared with the five previous seasons.

**Results:**

A dramatic reduction in influenza cases was seen in all three countries, with only a 3- to 6-week duration from the peak of weekly influenza cases until the percentage dropped below 1%. In contrast, in the previous nine influenza seasons, the decline from the seasonal peak to below 1% of influenza-positive samples took more than 10 weeks.

**Conclusions:**

The PHSM against SARS-CoV-2 were followed by a dramatic reduction in influenza cases, indicating a wider public health effect of the implemented measures.

## Introduction

Following the initial detection of the new severe acute respiratory syndrome coronavirus-2 (SARS-CoV‑2) in China in late December 2019, the coronavirus disease (COVID-19) spread rapidly to become a major global public health emergency [1,2]. In Europe, the first COVID-19 cases were reported in January 2020 and rapidly increased in the following weeks [3-5]. In Denmark, Norway and Sweden, laboratory testing for SARS-CoV‑2 was in place by end of January 2020 and the number of cases began to increase from early March (week 10). By early October 2020, ca 3.8 million cases and 193,000 deaths had been reported in the European Union/European Economic Area (EU/EEA) and the United Kingdom [6]. 

In Denmark and Norway, the most far-reaching COVID-19 preventive interventions were announced on 11 and 12 March 2020 (week 11), respectively, and implemented during the following days. The preventive measures included closure of day care centres, kindergartens, schools, and universities as well as cancellation of cultural events. People were also instructed to work from home if possible and keep physical distance. In Denmark, gatherings of more than 100 people were prohibited; in Norway, people were asked to avoid any unnecessary travel. 

**Figure 1:**
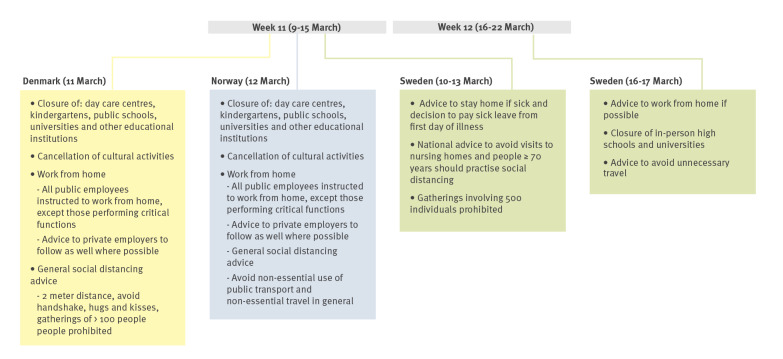
Interventions implemented to reduce the transmission of SARS-CoV-2 in Denmark, Norway and Sweden, 9–22 March 2020

In Sweden, recommendations and policy changes were announced around the time that the pandemic was declared (week 11–12). From week 11, people were advised to stay at home at any sign of symptoms, even mild ones. Of note, the sick leave was compensated by the state from the first day of illness, instead of after one mandatory qualifying day. People were also advised to avoid visiting nursing homes, people 70 years and older should practise physical distancing and gatherings of more than 500 people were prohibited. From week 12, people were asked to work from home and avoid unnecessary travel. High schools and universities were closed for in-person learning. The specific interventions in the three countries are shown in Figure 1.

SARS-CoV-2: sudden acute respiratory syndrome coronavirus-2.

These interventions were also expected to impact the transmission of other respiratory pathogens. In this report, we used data from the national influenza laboratory surveillance in the three countries to analyse the occurrence of influenza cases in the population during the early stages of the COVID-19 pandemic when the preventive measures were implemented [7].

## Methods

We compared data for the 2019/20 influenza season with data from five previous seasons in Denmark, Norway and Sweden. In all three countries, the occurrence of influenza in the population is monitored throughout the northern hemisphere influenza season (from week 40 of one year to week 20 of the following year) by the national public health institutes: the Danish Statens Serum Institut (SSI), the Norwegian Institute of Public Health (NIPH) and the Public Health Agency of Sweden. In all three countries, influenza surveillance is comprised of both a virological and an epidemiological component including laboratory surveillance, sentinel surveillance, surveillance of admissions to hospitals and intensive care units and registered mortality. The laboratory surveillance of influenza has been in place in all three countries for at least 10 years. 

In all three countries, the number of samples analysed for influenza, the number of laboratory-confirmed influenza cases detected per week and the proportion of influenza-positive samples are all used to monitor the influenza activity. The case definitions for influenza-like illness (ILI) from each country are presented (Box).

Box **Influenza-like illness definitions for Denmark, Norway, and Sweden**

**Denmark:** Sudden onset of symptoms and fever (≥ 38 ^o^C) and at least one of the following three respiratory symptoms: cough, sore throat or shortness of breath

**Norway:** Symptom start within the last 10 days, including sudden onset of respiratory symptoms, with fever (≥ 38 ^o^C) and cough

**Sweden:** Sudden onset of symptoms and at least one of the following four systemic symptoms: fever or feverishness, malaise, headache, myalgia and at least one of the following three respiratory symptoms: cough, sore throat or shortness of breath

In Denmark, national guidelines recommend that during the influenza season, patients in risk groups, including elderly people who present ILI to a general practitioner (GP) or with ILI and/or lower respiratory symptoms at a hospital, are tested for influenza virus (http://www.infmed.dk/guidelines). Electronic data on all patients swabbed at the GP or at hospitals and tested for influenza A and B viruses by PCR are registered in real time in the Danish Microbiology Database [8]. Each sample result provides information on date of sampling, if the sample was positive or negative for influenza virus and, in case of an influenza virus-positive test, whether influenza virus type A or B was detected.

In Norway, sentinel physicians throughout the country collect specimens from outpatients with ILI, which are sent for analysis at the NIPH-based National Influenza Centre (NIC). The sentinel samples are tested for influenza virus types A and B as well as the subtype by real-time PCR. In addition, laboratories perform influenza diagnostics on samples from GPs, while hospitals report the number of influenza virus-positive samples and the total number of specimens tested to the NIC on a weekly basis, according to virus type and subtype, detection method and patient age group. A selection of influenza-positive specimens is sent to the NIC for further characterisation.

In Sweden, influenza A and B cases are confirmed by PCR and reported in real time to the national surveillance database SmiNet in accordance with the Communicable Diseases Act [9]. All laboratories performing influenza diagnostics also voluntarily report denominator data i.e. the number of tests performed, weekly during the influenza season. Sampling for influenza diagnostics is performed following determination by the treating doctor based upon clinical indication. As such, many of the samples are from patients who have more severe symptoms or belong to risk groups and therefore require antiviral treatment or hospitalisation. Sentinel GPs also take samples from patients with acute respiratory infections and ILI, which are analysed to establish influenza type, subtype, and lineage by the Public Health Agency.

## Results

### Influenza season 2019/20

In all three countries, the 2019/20 influenza season was reported as a mild season; influenza A and B viruses were co-circulating with an increased number of cases by mid-December. Influenza A(H3N2) virus was the dominant subtype with clade 3C.3a (vaccine strain) circulating in all three countries, while clade 3C.2a1b was co-circulating in Denmark and Sweden, and 3C.2a1b was the most dominant clade in Norway. B/Victoria was the dominating influenza B virus lineage in all three countries. 

**Figure 2:**
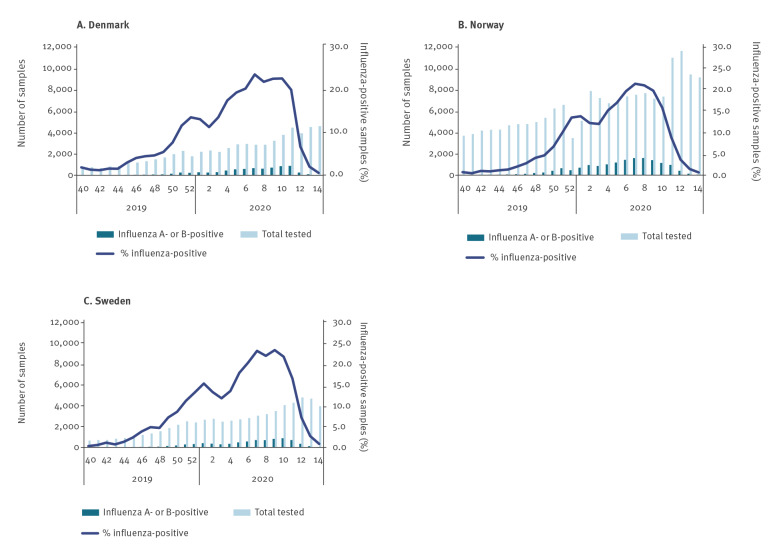
Laboratory-confirmed influenza A and B in Denmark, Norway and Sweden, 30 September 2019–5 April 2020

The percentage of influenza-positive samples exceeded 20% in week 6 in Denmark and Sweden and in week 7 in Norway. The percentage of positive samples remained above 20% for 2 to 5 weeks, followed by a prompt decline from week 11 to 12 in Denmark and Sweden and from week 10 to 11 in Norway. In Denmark, the percentage of influenza-positive samples decreased from 20% in week 11 to 0.6% in week 14; in Sweden, from 17% in week 11 to 0.8% in week 14; in Norway, from 16% in week 10 to 0.6% in week 14 (Figure 2). In Denmark and Sweden, the number of weekly influenza samples analysed increased slightly from week 9 and onwards. In Norway, a substantial increase was observed in the number of samples tested (7,315 in week 10 to 10,942 in week 11). In all three countries, a decrease in the percentage of influenza-positive samples was observed in week 2 and 3 in 2020.

### Impact of preventive measures against COVID-19 on seasonal influenza

**Figure 3:**
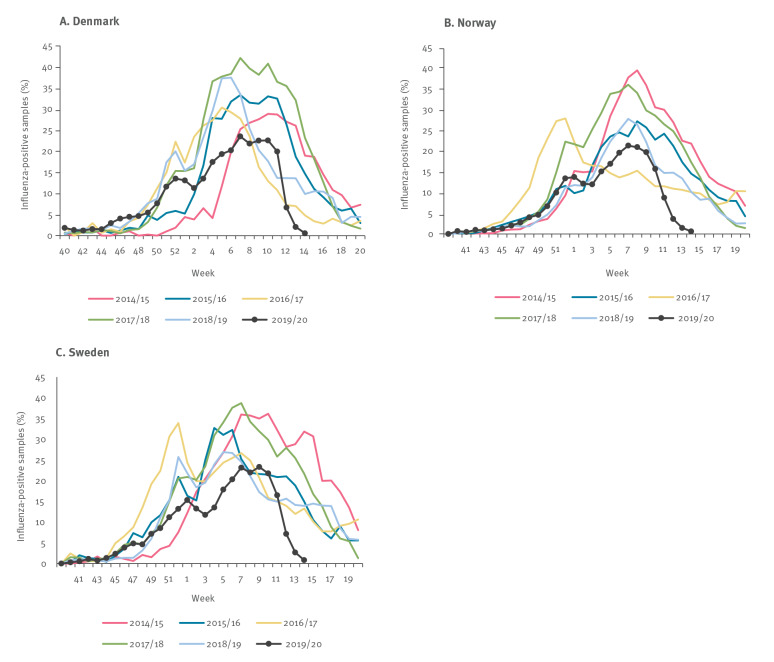
Percentage of influenza-positive samples per week in Denmark, Norway and Sweden, influenza seasons 2014/15–2019/20

A comparison of the 2019/20 influenza season with the previous five influenza seasons in each country reveals a marked drop in percentage influenza-positive samples in the 2019/20 season compared with the previous five influenza seasons (Figure 3). In the 2019/20 influenza season, the duration from the peak (highest number of registered weekly influenza-positive samples) until the percentage of influenza-positive samples was below 1% (week 14) was only 3 to 6 weeks in all three countries. In contrast, in the previous nine influenza seasons, the decline from the seasonal peak to below 1% of influenza-positive samples took more than 10 weeks (data not shown). Of note, in only one of nine previous seasons was the percentage of influenza-positive samples below 1% by week 20 i.e. when the influenza season officially ends.

## Discussion

We have shown that the number of laboratory-confirmed influenza cases in Denmark, Norway and Sweden decreased dramatically, despite increased testing, from week 10 in 2020 and onwards during the period when SARS-CoV-2 preventive measures were implemented. A similar marked decline in laboratory-confirmed influenza has not been observed previously during at least the past decade in Denmark, Norway and Sweden. This decline stands out from the previous nine influenza seasons, where the duration from the peak until the percentage of weekly cases was below 1% lasted more than 10 weeks as compared with only 3 to 6 weeks in the 2019/20 season. A similar impact was observed in other European countries e.g. Austria, Belgium, Germany, Italy, the Netherlands and Spain [10] where restrictions on schools, workplace activities and mass gatherings were also implemented [10]. This strongly suggests that the preventive measures taken against the spread of SARS-CoV-2 have also interrupted the spread of influenza.

In Denmark, Norway and Sweden, the number of people who tested positive for SARS-CoV-2 started to increase on 8 March, 9 March and 3 March 2020, respectively. In Denmark and Norway, patients with COVID-19 symptoms were encouraged to call their GP or use online healthcare consultations for decisions on being tested at a medical facility. In Sweden, people were recommended to call the medical advice line 1177 before seeking healthcare. 

Since COVID-19 symptoms are similar to influenza symptoms, it is probable that individuals referred to hospitals with COVID-19 symptoms were routinely tested for influenza in all three countries. In addition, individuals who would not normally seek medical help with influenza-like symptoms might be more likely to contact the GP because of concerns about COVID-19. This can explain why the number of tests for influenza continued to increase despite very few samples testing positive. From week 10 to 11, a 50% increase in number of samples tested for influenza was observed in Norway, which coincided with a decrease in percentage influenza-positive samples already from week 10 to week 11. This explains the decrease in percentage positive influenza samples observed just before the interventions were introduced.

Denmark and Norway introduced the most extensive interventions during week 11, while Sweden introduced interventions in two steps during week 11 and 12. In Norway and Denmark, all day care centres, kindergartens and schools were closed, which was not the case in Sweden. In addition, physical distancing was advised for all ages in Denmark and Norway, not only for those above 70 years of age as in Sweden. People were advised to work from home one week later in Sweden as compared with Denmark and Norway. On the contrary, Sweden had national recommendations to avoid visits to nursing homes from week 11, which was not the case in Denmark and Norway. Although the interventions and timing introduced by the three countries differed, a similar decline in laboratory-confirmed influenza was observed from week 11 to 12 in Denmark and Sweden and from week 10 to 11 in Norway. Based on the observations from the three countries, it is not possible to conclude whether one intervention compared to another was more effective in eliciting a reduction in influenza. It is probable that the increase in COVID-19 cases in week 10 and 11, coupled with the concern about a new disease in the general population, led to some degree of social distancing in all age groups, which may have contributed to the interruption of the influenza transmission.

We also observed a slight reduction of influenza-positive samples during week 2 and 3. Christmas and New Year holidays represent a common annual period when day care centres, schools and universities are closed, fewer people are working and the public transportation system is less crowded. This could explain the decrease in percentage influenza-positive samples during week 2 and 3. A similar decrease was also observed in previous influenza seasons when the circulation of influenza started before Christmas.

The recommendations for vaccination against influenza during the 2019/20 season did not change in any of the countries compared with previous seasons. The influenza vaccines are normally administered from 1 October each year. However, in the 2019/20 season, the influenza vaccination was delayed 2 to 4 weeks because of a later than usual selection of the A(H3N2) vaccine strain by the World Health Organization. Of note, in Denmark and Norway, the vaccine administered was a quadrivalent inactivated vaccine, while in the previous season, trivalent and quadrivalent inactivated vaccines were offered. In Sweden, the quadrivalent inactivated vaccine was administered in both the 2018/19 and 2019/20 seasons. However, it is unlikely that a late vaccination start or the change in type of vaccine could have resulted in the sudden sharp decline seen in the influenza transmission mid-March, 2020. In addition, it may be speculated that an interaction between infection with influenza and SARS-CoV-2 within individuals could have led to the decline in influenza. However, influenza had already circulated for several weeks by the time SARS-CoV-2 was introduced and a containment strategy was established to minimise the spread of SARS-CoV-2 in the three countries. Therefore, it is not likely that immunity among relatively few SARS-CoV-2-infected individuals could stop the circulation of influenza within the population. In the southern hemisphere, there was almost no influenza circulating during the winter 2020 [11]. 

The same scenario occurred in the 2020/21 season in the northern hemisphere [10]. Depending on how widespread the PHSM will be during the 2021/22 season, we might observe an additional influenza season with limited virus circulation; however, a rebound season with higher levels cannot be ruled out. With low or no circulation of influenza viruses for one or two seasons, young children are not exposed and a larger group of children will be susceptible in the following influenza seasons.

We have shown how the PHSM against COVID-19 affected the occurrence of influenza in Denmark, Norway and Sweden, which is supported by similar findings in Hong Kong and the United States [11,12]. In addition, the enhanced focus on improved hand hygiene that has come from the COVID-19 measures could have further prevented transmission of the influenza as well as other infections. However, further studies are needed to show the full impact of the PHSM on public health. 

This is an ecological study based on PHSM against COVID-19 coinciding with the reduction in the spread of influenza. As such, this study cannot measure the effect of different PHSM on the spread of influenza or conclusively determine a causal link, but can point to a seemingly strong effect that is epidemiologically plausible. 

In conclusion, the implementation of PHSM against COVID-19 in the Scandinavian countries resulted in a marked decline in transmission of influenza and termination of the influenza season much sooner than expected. This indicates that the PHSM and the subsequent population behaviour change were a very powerful tool, although we do not know which interventions had most impact. In the 2020/21 influenza season, no influenza epidemic was seen in the Scandinavian countries or the rest of the Northern Hemisphere, which indicates that ongoing PHSM, nationally and globally, in combination with other pandemic changes such as reduced global travel, continued to affect the spread of influenza. If some PHSM are maintained in the future, this might influence future influenza epidemics.
